# Visual processing deficits in patients with schizophrenia spectrum and bipolar disorders and associations with psychotic symptoms, and intellectual abilities

**DOI:** 10.1016/j.heliyon.2023.e13354

**Published:** 2023-02-01

**Authors:** Aili R. Løchen, Knut K. Kolskår, Ann-Marie G. de Lange, Markus H. Sneve, Beathe Haatveit, Trine V. Lagerberg, Torill Ueland, Ingrid Melle, Ole A. Andreassen, Lars T. Westlye, Dag Alnæs

**Affiliations:** a NORMENT, Division of Mental Health and Addiction, Oslo University Hospital & Institute of Clinical Medicine, University of Oslo, Norway; b Sunnaas Rehabilitation Hospital HT, Nesodden, Norway; c Department of Psychology, University of Oslo, Norway; d LREN, Centre for Research in Neurosciences, Department of Clinical Neurosciences, Lausanne University Hospital (CHUV) and University of Lausanne, Lausanne, Switzerland; e Department of Psychiatry, University of Oxford, Oxford, UK; f KG Jebsen Centre for Neurodevelopmental Disorders, University of Oslo, Norway; g Kristiania University College, Oslo, Norway

**Keywords:** Visual discrimination, Spatial frequency, Bipolar disorder, Schizophrenia, Mental illness, Early sensory processing

## Abstract

**Objective:**

Low-level sensory disruption is hypothesized as a precursor to clinical and cognitive symptoms in severe mental disorders. We compared visual discrimination performance in patients with schizophrenia spectrum disorder or bipolar disorder with healthy controls, and investigated associations with clinical symptoms and IQ.

**Methods:**

Patients with schizophrenia spectrum disorder (n = 32), bipolar disorder (n = 55) and healthy controls (n = 152) completed a computerized visual discrimination task. Participants responded whether the latter of two consecutive grids had higher or lower spatial frequency, and discrimination thresholds were estimated using an adaptive maximum likelihood procedure. Case-control differences in threshold were assessed using linear regression, F-test and post-hoc pair-wise comparisons. Linear models were used to test for associations between visual discrimination threshold and psychotic symptoms derived from the PANSS and IQ assessed using the Matrix Reasoning and Vocabulary subtests from the Wechsler Abbreviated Scale of Intelligence (WASI).

**Results:**

Robust regression revealed a significant main effect of diagnosis on discrimination threshold (robust F = 6.76, p = .001). Post-hoc comparisons revealed that patients with a schizophrenia spectrum disorder (mean = 14%, SD = 0.08) had higher thresholds compared to healthy controls (mean = 10.8%, SD = 0.07, β = 0.35, t = 3.4, p = .002), as did patients with bipolar disorder (12.23%, SD = 0.07, β = 0.21, t = 2.42, p = .04). There was no significant difference between bipolar disorder and schizophrenia (β = −0.14, t = −1.2, p = .45). Linear models revealed negative associations between IQ and threshold across all participants when controlling for diagnostic group (β = −0.3, t = −3.43, p = .0007). This association was found within healthy controls (t = −3.72, p = .0003) and patients with bipolar disorder (t = −2.53, p = .015), and no significant group by IQ interaction on threshold (F = 0.044, p = .97). There were no significant associations between PANSS domain scores and discrimination threshold.

**Conclusion:**

Patients with schizophrenia spectrum or bipolar disorders exhibited higher visual discrimination thresholds than healthy controls, supporting early visual deficits among patients with severe mental illness. Discrimination threshold was negatively associated with IQ among healthy controls and bipolar disorder patients. These findings elucidate perception-related disease mechanisms in severe mental illness, which warrants replication in independent samples.

## 1 Introduction

Schizophrenia and bipolar disorder are severe mental disorders characterized by perceptual, cognitive and emotional symptoms [1]. Perceptual disruptions across several domains are defining features of schizophrenia, but are also frequently reported in bipolar disorder [2], both in manic and depressive phases [3]. Previous studies of patients with schizophrenia have reported disruptions in speech [4], object [5] and face [6] perception, as well as in basic sensory and perceptual processes [7], including tone-matching, contrast sensitivity, and spatial frequency discrimination.

Low-level sensory and perceptual mechanisms enable the organism to detect, discriminate and categorize basic sensory features, such as pitch and tone discrimination in the auditory modality, and orientation, spatial frequency and colors in the visual domain [[8], [9], [10]]. These functions reflect partly automated “bottom-up” cortical processing, and constitute the early stages of the sensory and perceptual streams in the visual processing hierarchy [11]. These building blocks give rise to higher-level perceptual abstractions such as objects, faces, location and movement [12,13]. Importantly, this complex integrative process has been shown to also involve higher-level cognition or “top-down” executive functions such as decision making, memory, planning, [14,15]. Disruptions in these early sensory and perceptual pathways are hypothesized to constitute precursors to the abnormal higher-level abstract perceptual experiences (e.g., hallucinations) and beliefs (e.g., delusions) often reported by patients with severe mental illness [[16], [17], [18]].

The relevance of visual discrimination performance and sensory impairments for mental illness is supported by several lines of evidence. A prospective study of children of mothers with schizophrenia revealed that visual, but not auditory dysfunction at age four was predictive of schizophrenia in adulthood [19]. Schizophrenia has been associated with impaired performance on a range of visual discrimination and recognition tasks, such as patterns, locations, movement trajectories and spatial frequency [20], contrast sensitivity [21,22], and lower susceptibility to visual illusions compared to healthy controls [23,24]. Visual perceptual abnormalities were reported to be more predictive of conversion from a psychosis prodrome to schizophrenia than symptoms of thought and language problems and ideas of reference [25], and are linked to functional outcome in schizophrenia [26,27]. Impaired visual processing has also been linked to transdiagnostic vulnerability in mental illness, often termed P or general psychopathology [28]. Recent studies have reported that individual differences in P are linked to aberrant structure [29] and functional connectivity of visual cortical areas of the brain [30]. This underscores the clinical relevance of assessing early and low-level perceptual processes in severe mental illness.

Cognitive impairments are often reported among patients with schizophrenia [31], and are also found in bipolar disorder [32,33]. At the group level, patients with schizophrenia show reduced performance across several cognitive domains, including speed of processing, attention, vigilance, working memory, verbal learning and memory, visual learning and memory, reasoning and problem solving and verbal comprehension [[34], [35], [36], [37]]. Perceptual disruptions have been attributed to impaired executive functions such as flexible shifting of attention, inhibitory control and working memory [38,39], and aberrant early sensory and perceptual processing may contribute to the clinical and cognitive symptoms in schizophrenia.

Here, using a computerized discrimination threshold estimation task along the basic visual sensory dimension of spatial frequency, we compared visual discrimination performance in patients with schizophrenia spectrum or bipolar disorders to healthy controls. In addition to testing for case-control differences, we investigated associations between discrimination threshold and positive and negative symptoms and IQ. Based on current theories and the reviewed literature we hypothesized that patients with severe mental disorders would exhibit higher discrimination thresholds, indicating worse performance, compared to healthy controls. Our final hypothesis is based on the notion of a hierarchical processing stream in which early sensory mechanisms represent precursors to more abstract and complex functions. We anticipated that higher visual discrimination threshold would be associated with more severe clinical symptoms as measured using the positive and negative syndrome scale (PANSS) and lower IQ.

## 2 Materials and methods

### 2.1 Sample and exclusion criteria

A total of 165 healthy controls (HC) and 83 patients with severe mental disorders completed the task, including schizophrenia (n = 28), schizophreniform (n = 1), schizoaffective disorder (n = 4), bipolar I disorder (n = 25) and bipolar II disorder (n = 30). Patients with schizophrenia, schizophreniform or schizoaffective disorders were combined into a schizophrenia spectrum disorders group (SZ, n = 33), and patients with bipolar disorder I or bipolar disorder II were combined into a bipolar spectrum disorders group (BD, n = 55). After exclusion based on task performance (see below and Supplementary Fig. S1), 32 patients with SZ, 54 patients with BD and 148 HC were included in the analysis. Table 1 summarizes demographics and clinical variables. We assessed covariate balance by estimating standardized mean differences [40] for SZ and BD versus HC, yielding the following results: SZ: IQ = −0.91, age = −0.71, sex = 0.05; BD: IQ = -0.26, age = −0.45, sex = 0.46. Distribution of age, IQ and education are shown in Supplementary Fig. S2. The percentages of missing data were the following for each variable across all patients: 2.33% missing education mean years, 6.98% for mean IQ estimates, 0% for threshold estimations, age and sex, 4.65% for PANSS negative and general mean scores and 3.49% for PANSS positive mean scores. Specifically, there were two cases in the SZ group missing data on IQ and PANSS scores, and two cases of BD patients missing data on PANSS, four BD patients missing data on mean IQ scores.

**Table 1:** Demographics and clinical characteristics including the subscales and total symptom score from PANSS for schizophrenia spectrum disorders (SZ), bipolar disorder (BD), and healthy controls, (HC). The Intelligence Quotient (IQ) was estimated from the Wechsler Abbreviated Scale of Intelligence (WASI).

	HC	BD	SZ
N	148	54	32
Age Mean (SD) years	39.9 (9.6)	33.8 (13.1)	33.7 (8.54)
Sex (% female)	44.5	64.8	50
Education Mean (SD) years	14.9 (2.1)	14.5 (2.24)	13.7 (2.71)
Discrimination threshold Mean (SD)	0.11 (0.07)	0.12 (0.07)	0.14 (0.08)
PANSS Positive Mean (SD)	–	9.04 (3.04)	14.7 (4.89)
PANSS Negative Mean (SD)	–	9.52 (2.93)	14.0 (6.24)
PANSS General Mean (SD)	–	25.7 (5.47)	27.2 (6.42)
PANSS Total Mean (SD)	–	44.3 (9.54)	49.5 (13.7)
IQ Mean (SD)	116 (9.06)	113 (9.08)	104 (12.6)

Patients were recruited from psychiatric hospitals and outpatient clinics in the Oslo region, Norway, as part of the ongoing Thematically Organized Psychosis (TOP) study. Healthy controls from the same geographic area were identified by a stratified random draw from the national population registry and invited by letter. They were screened and excluded if they had experienced any psychiatric disorder, or their first-degree relatives had experienced a severe psychiatric disorder. Exclusion criteria included age <18 years, IQ < 70, previous moderate or severe head injury or a neurologic illness. All participants gave written informed consent before participating. The study was approved by the Regional Committee of Medical Research Ethics and the Norwegian Data Inspectorate and was conducted in accordance with the Helsinki declaration.

### 2.2 Clinical and cognitive assessment

Diagnoses were assessed by clinical psychologists or physicians using the Structured Clinical Interview for DSM-IV axis I disorders (SCID-I) modules A-E [41]. Diagnostic reliability has previously been found to be satisfactory [42]. Patients meeting criteria for DSM-IV axis diagnoses of schizophrenia spectrum disorders (schizophrenia, schizoaffective, schizophreniform) or bipolar disorder (Bipolar type I or II) were included, thus excluding participants diagnosed with non-specified mental disorders (including single-episode psychotic disorders, non-specified affective disorders).

Current psychotic symptoms were assessed with the Positive and Negative Syndrome Scale [PANSS; 43], which is widely used in clinical and research settings and considered a reliable means of symptom assessment [44,45]. Medication was reported and grouped as antipsychotics, antidepressants, antiepileptics or mood stabilizers according to the guidelines from the World Health Organization Collaborating Center for Drug Statistics Methodology (https://www.whocc.atcdd). The defined daily dose (DDD) is the assumed average maintenance dose per day for a drug used for its main indication in adults, and mean usage is the DDD per user per day. In our patient sample of BD patients, the mean DDD for antipsychotics for was 0.108, for antidepressants 0.535, for antiepileptica 0.056, 0.024 for mood stabilizers (Litium). With regards to SZ, the mean DDD for antipsychotics was at 0.364, 0 for antiepileptica, 0.33 for antidepressants and 0 for Litium. To summarize, the mean medications total DDD was estimated at 1.11 for the SZ subgroup and 0.69 for the BD subgroup. Positive symptoms refer to an excess or distortion of normal functions (e.g., hallucinations and delusions), while negative symptoms represent a diminution or loss of normal functions. Estimated IQ scores were assessed using the Matrix Reasoning and the Vocabulary subtests from the Wechsler Abbreviated Scale of Intelligence, WASI [46]. Bipolar patients were euthymic at the time of examination.

Participants were excluded based of the following criteria: an estimated threshold >100% difference, meaning more than a doubling or halving of the spatial frequency of the test stimulus relative to the sample, on any of the four rounds. Consequently, six participants were excluded (4 HC, 1 patient with SZ, 1 patient with BD). Supplementary Fig. S1 shows a flow chart of the exclusion and inclusion of participants.

### 2.3 Stimulus presentation

**Fig. 1:**
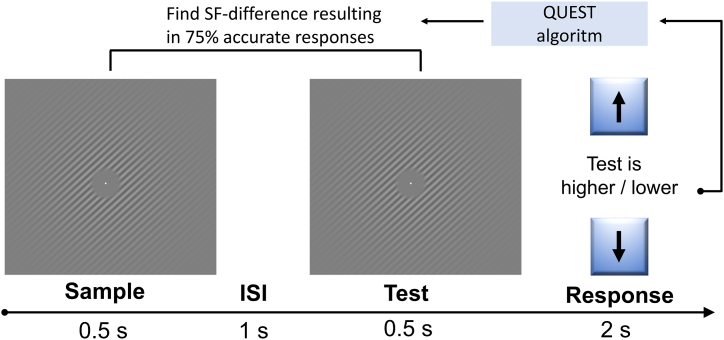
The visual discrimination task. Participants performed 4 rounds of a 2-alternative forced choice task where they indicated whether the test grating had a higher or lower spatial frequency compared to the sample stimulus, briefly interrupted by an inter-stimulus interval (ISI).

Fig. 1 illustrates the experimental paradigm. The task was adapted from Sneve, Alnæs [47], and consisted of sinusoidal grating patterns with varying spatial frequency. Each round of the task consisted of 32 trials, and each trial consisted of 0.5 s presentation of a sample grating, followed by a 1 s inter-stimulus interval (ISI), and then a 0.5 s presentation of the test grating, after which the participants indicated whether the test had a higher or lower spatial frequency compared to the sample by pressing the up or down-arrow on the keyboard (2-alternative forced choice). Each participant completed 4 rounds of the task. Participants were seated approximately 50–60 cm from the laptop screen (screen width 30.5 cm). The sinusoid had a maximum Michelson's contrast of 0.6, tapered with a Gaussian kernel with a standard deviation of 2.5. Base cycles per visual degrees were set to vary between 2, 3 and 4 across trials, while orientations of the spatial frequency grids varied with 90° across trials.

### 2.4 Visual discrimination threshold estimation

The paradigm was coded and run using Psychtoolbox-3 [48] in MATLAB (version 2015a; Mathworks, Natick, Massachusetts). Individual 75% discrimination thresholds were estimated using an adaptive maximum likelihood procedure [QUEST]; [49]. The QUEST algorithm varied the spatial frequency difference of the test stimulus compared to the sample stimulus, converging on the difference producing a discrimination accuracy of 75%. This threshold is selected based on the proportion of correct forced-choice responses as a function of each level of the physical stimulus, a principle regularly used in sensory detection and sensory discrimination tasks called the psychometric function. If the stimulus difference is large enough, participants will be able to answer 100% of the trials correctly, while if the stimulus difference is sufficiently small, the task will be impossible to solve. A discrimination threshold of 75% is used in this study in order to find the point at which the participants have an above-chance rate of correct responses while not always responding correctly, i.e. the point at which a change in stimulus will have the greatest effect on task accuracy and thus be the most sensitive area to detect differences in discrimination ability. All participants completed 4 rounds of 32 trials. Each round took approximately 2 min to complete. The first estimation round always started using the same spatial frequency difference across participants, while subsequent runs used the previous estimated difference threshold as the updated initial value. Supplemental Fig. S3 shows the bivariate correlation of estimated thresholds between the four rounds. Individual average threshold levels were then calculated as the average across the final three rounds.

### 2.5 Statistical analyses

Statistical analyses were performed using R (version 3.5.1, https://www.r-project.org). Bivariate correlational analyses were performed to investigate whether threshold estimations converged across sessions. For group comparisons we employed robust linear regression (Threshold ∼ Diagnosis + Age + Sex, using “lmr” from the R package ‘MASS’ [50], followed by a robust F-test (“f.robustftest” from the R package ‘sfsmisc’ [51] and post-hoc comparisons of group differences corrected using Tukey (using “glht” from the R-package ‘multcomp’ [52]. Robust regression weights data points to reduce the impact of outliers and noisy observations, while still including all the data in the analysis. We also performed non-parametric group comparisons using Wilcoxon rank sum tests for independent groups. For transparency, we also report the results from a standard linear regression and the accompanying post-hoc tests.

Finally, we tested for associations between visual discrimination threshold and PANSS domain scores and IQ using separate multiple linear regression analyses within groups (PANSS only available from patients), with age and sex as covariates (*Threshold ∼ Age + Sex + Cognition/Clinical*). We tested effects of IQ both using the global IQ estimate and separating the visuospatial matrices scores and the vocabulary tasks. Clinical symptom scores were calculated using PANSS subscales, separating positive, negative and generalized symptoms [43,53]. Compared to healthy controls, IQ was significantly lower among patients with schizophrenia spectrum disorder (β = −1.06, t = −5.36, p = .5.3e-08), but not among patients with bipolar disorder (β = −0.17, t = −1.11, p = .27). Since our design does not allow us to disentangle the effects of IQ from diagnosis, we investigated associations between visual discrimination performance and IQ within groups.

## 3 Results

### 3.1 Group differences in visual discrimination threshold

**Fig. 2:**
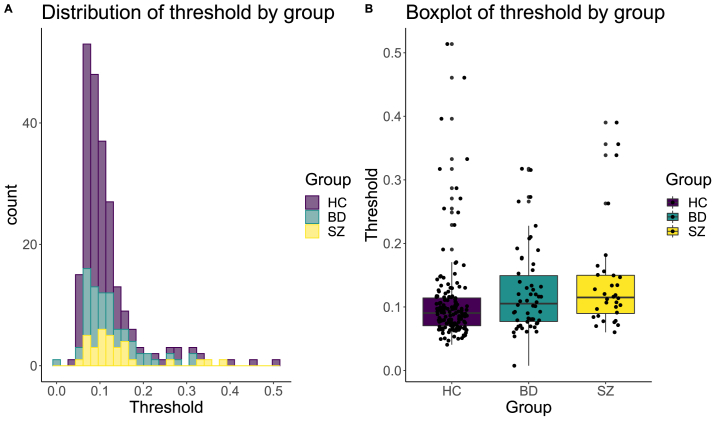
A) The distribution of visual discrimination thresholds by diagnostic group. B) Boxplot of visual discrimination threshold by diagnostic group. Robust regression revealed significant differences in threshold for both schizophrenia (SZ) and bipolar (BD) patients vs healthy controls (HC**)**, while no difference was found for SZ vs BD.

Fig. 2 A and B shows a histogram and a boxplot, respectively, of the estimated thresholds by group. Robust regression revealed a significant effect of diagnosis on discrimination thresholds (robust F = 6.76, p = .001). Post-hoc comparisons corrected for multiple comparisons using Tukey revealed that patients with SZ (mean = 14%, SD = 0.08) had significantly higher estimated discrimination thresholds than healthy controls (mean = 10.8%, SD = 0.07, β = 0.35, t = 3.4, p = .002), as did patients with BD (mean = 12.3%, SD = 0.07, β = 0.21, t = 2.42, p = .04). There was no significant difference between BD and SZ (β = −0.14, t = −1.2, p = .45). Non-parametric group comparisons using Wilcoxon rank sum revealed significant differences between HC compared to SZ (p = .001) and BD (p = .035), and no significant difference between SZ and BD (p = .28).

The standard linear regression (Threshold ∼ Diagnosis + Age + Sex) showed a significant main effect of diagnosis (F = 3.6, p = .03). Post-hoc pairwise group comparisons corrected for multiple comparisons using Tukey revealed significantly higher threshold among patients with SZ compared to HC (β = 0.49, t = 2.5, p = .03), and no significant differences between patients with BD HC (β = 0.25 t = 1.56, p = .26), nor between the two patient groups (β = −0.24, t = −1.09, p = .52).

### 3.2 Associations between visual discrimination performance and IQ

**Fig. 3:**
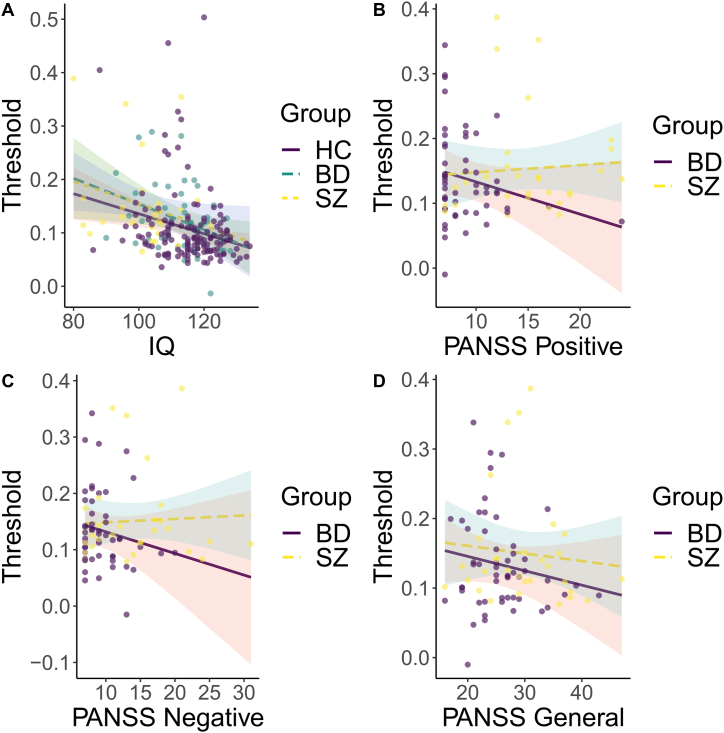
There was no significant IQ by group interaction. For within-group analysis, IQ showed a significant association with discrimination threshold. There were no significant associations of any of the PANSS scores with discrimination threshold. (A) Discrimination thresholds plotted against IQ, by group. (B) Discrimination thresholds plotted against positive PANSS scores, by diagnosis (C) Discrimination thresholds plotted against negative PANSS scores, by diagnosis. (D) Discrimination thresholds plotted against general PANSS scores, by diagnosis.

Fig. 3A shows the relationship between discrimination threshold and IQ. Linear models across all participants revealed a significant negative association between visual discrimination threshold and IQ when controlling for diagnostic group (β = −0.3, t = −3.43, p = .0007). Within groups, a significant negative association was found among healthy controls (β = −0.29, t = −3.72, p = .0003) and patients with bipolar disorder (β = −0.34, t = −2.53, p = .015), but not among patients with schizophrenia spectrum disorder (β = −0.22, t = −1.34, p = .19). Separating domain scores on the WASI revealed that the association between threshold and IQ were explained by differences on the visuospatial matrices task for healthy controls (β = −0.32, t = −3.5, p = .001) and bipolar patients (β = −0.35, t = −3.04, p = .004), while the vocabulary domain scores revealed no significant associations for healthy controls (β = −0.09, t = −0.955, p = .34), and bipolar patients (β = −0.13, t = −96, p = .344). Follow-up analysis revealed no significant interaction effect for diagnostic group on IQ (F = 0.044, p = .97).

### 3.3 Associations between visual discrimination performance and PANSS symptom domains

Fig. 3B–D depict the associations between PANSS domain scores and visual discrimination threshold. Linear models within patients with SZ revealed no significant association between discrimination threshold and the PANSS positive (β = 0.104, t = 0.58, p = .6), negative (β = −0.01, t = 0.046, p = .96) and general (β = −0.08, t = −0.5, p = .64) subscales. For patients with BD, no significant associations were found for the PANSS positive (β = −0.24, t = −1.222, p = .227), negative (β = −0.23, t = −1.09, p = .281), nor the general (β = −0.21, t = −1.37, p = .178) subscales.

## 4 Discussion

Patients with severe mental illnesses including schizophrenia spectrum and bipolar disorders often experience neurocognitive deficits across several domains. Here, we show that patients with schizophrenia spectrum or bipolar disorders have higher visual discrimination thresholds for the basic visual feature of spatial frequencies compared to healthy controls. This is consistent with previous reports of basic visual processing deficits in patients with severe mental disorders [54,55]. Overall, the results support the notion of disrupted basic visual processing in patients with schizophrenia [22,56,57], and are in line with previous reports of visual discrimination deficits in patients with bipolar disorder [2].

Patients with schizophrenia and bipolar disorders are heterogeneous both at the clinical, cognitive and brain biological level [[58], [59], [60], [61]]. Some of this clinical heterogeneity may be resolved by combining clinical profiling with detailed neurocognitive assessment across the hierarchical processing streams from early sensory and perceptual mechanisms to later and more abstract processes including decision making and reasoning. In the case of the current study, we found no association with the level of psychotic symptoms.

Disrupted early visual processing in patients with severe mental illness has also been linked to dysfunction in neurotransmission systems [62], with reduced visual cortical GABA concentration associated with aberrant surround suppression in patients with schizophrenia [63,64]. With regards to everyday life, early visual processing deficits may influence psychosocial and daily functioning through their impact on patients' ability to focus, encode relevant information [65], and directing attention toward relevant target stimuli in the surroundings. Being able to both extract and focus on relevant information and joint attention with people in the surroundings is important for both working life and psychosocial functioning [66,67], as well as patients daily living autonomy [68]. Impaired neurocognition, including visual sensory disruption in severe mental disorders, is often present before disease onset and persists over time, but is also linked to illness state [69,70], and is affected by antipsychotics medication [71]. Visual discrimination deficits in SZ have been reported also in high functioning remitted and unmedicated patients [72], and visual perceptual disturbances are predictive of conversion to psychosis in prodromal individuals [25], supporting visual processing disruptions as a trait preceding illness onset. The current study design does now allow us to disentangle preceding trait effects from disease and medication effects, and the reported differences likely reflect a mix. Early visual processing impairments could be linked to the underlying vulnerability for mental illness, and possibly serve as a proxy of impaired basic brain functions, rather than simply resulting from higher-order deficits of attention and executive function [73]. Studies investigating the role of visual perceptual processing and associations with general risk factors for mental illness in childhood and adolescence are therefore warranted.

Our analysis revealed negative associations between IQ and visual discrimination thresholds across all participants, and specifically within HC and BD patients. Importantly, this association seemed to be driven by differences on the non-verbal cognitive tasks of the WASI. Power-limitations may explain why we could not establish this relationship in our SZ sample, as early visual processing as precursors to higher-order cognitive functioning are frequently reported in the literature [[74], [75], [76], [77]], and the notion of early visual processing as precursors of higher-order cognitive functioning. Our results did not show any significant associations between discrimination thresholds and symptom severity. There is always the possibility that we were not able to detect a true relationship due to the relatively small variation in PANSS scores in our sample, resulting in a type II error. Previous studies have reported associations between impaired sensory discrimination and poor cognitive functioning in people with psychosis that are not solely explained by their psychiatric symptoms [78]. It has been suggested that sensory disruptions, for example in motion processing, are due to higher-order memory processing deficits, caused or influenced by existing disruptions in top-down processes [79]. Future studies may be able to investigate the integration between such top-down and bottom-up processes and its relation to the observed cognitive deficits in patients with severe mental disorders.

Some studies have reported associations between symptom severity measured with the PANSS and performance on an auditory tone matching task, where the mismatch-negativity (MMN) as measured using EEG [80] was inversely associated with right-ear performance on a dichotic listening task [81] and performance on a face recognition task [82]. In contrast, our analysis revealed no significant associations between PANSS domain scores and visual discrimination thresholds, and our findings are thus in line with studies reporting no robust associations between visual performance and symptom load [54,83].

Of interest to future studies and potential for clinical implementation, performance on sensory discrimination is prone to training effects [84], termed perceptual plasticity, and robust plasticity has been reported for the visual system in patients with schizophrenia [85]. This could in principle be used to develop bottom-up behavioral strategies as a part of an intervention or treatment protocol. Future investigations may also assess the relationships between sensory discrimination deficits across auditory and visual modalities, as auditory perceptual disruptions are reported as more frequent. Previous studies have suggested a link between the two [78].

The current findings should be interpreted in light of some limitations. Effects of medication were not explicitly tested for in this study. Previous studies have suggested that medication may partly explain mixed results on behavioral, perceptual and cognitive deficits among patients with severe mental illness. Our design did not allow us to rule out medication effects on visual discrimination ability [86]. Investigating effects of medication requires experimental control of medication type and doses, and was therefore not feasible in the current study. The current experimental task was employed as part of a brief assessment prior to brain MRI assessment. While demonstrating feasibility in a clinical setting, stricter experimental control of variables such as ambient light, visual acuity and distance to screen as afforded by a psychophysics lab environment would also be beneficial for future studies employing similar psychophysical methods. The interval of 1 s between sample and test stimuli in the paradigm makes it difficult to disentangling differential disruptions in encoding and retention of stimuli, reflecting separate but interacting processes in visual working memory, involving overlapping neural systems [87].

Also, patient sample sizes and thereby statistical power could have affected our ability to probe the relationship between visual perception and clinical symptoms. Cognitive deficits in bipolar disorders are frequently reported, but typically less severe than those observed for schizophrenia [88]. All our groups had mean IQ levels which are nominally larger than the population average, indicating that our participants represent a relatively cognitive high-functioning part of the population. Also, the PANSS scores for our samples were generally low. These characteristics must be taken into consideration regarding the generalizability of our results to the clinical and healthy population at large. Domain-scores from the full-scale Wechsler Adult Intelligence Scale (WAIS) may reveal more specific associations between cognitive domains and visual discrimination impairments. Also, our cross-sectional design did not allow us to disentangle trait effects from disease-related state effects on visual discrimination. Future studies may investigate a broader range of the clinical severity spectrum. Further, the difference between patients with bipolar disorder and healthy controls was significant using robust linear regression, but not when using a standard linear model, suggesting outliers may mask group differences. With regards to ethical considerations, it is worth mentioning that studies that investigate early predictors of psychosis development may increase our ability to predict which individuals are at risk of developing a severe mental illness. In the ethical literature on severe mental illness, the possible adverse effects of early diagnosis and treatment and discovering patient's individual levels of risk have been largely ignored [89]. Future initiatives should also consider systematically assessing attitudes and needs of patients in conjunction with the advancements in science and technology.

In conclusion, our findings of early visual processing deficits in patients diagnosed with schizophrenia spectrum or bipolar disorders, suggest an association between early visual processing and general intellectual abilities in patients with bipolar disorder and healthy controls. Taken together, these findings elucidate underlying disease mechanisms in severe mental illness. Future studies investigating brain mechanisms associated with such basic sensory deficits, including functional brain imaging and mapping of neurotransmitter function in individual patients have the potential to yield important insights into the neurocognitive mechanisms of psychotic disorders.

## Author contribution statement

A.R.L, K.K.K., L.T.W., M.H.S., and D.A. conceived and designed the experiments; A.R.L. performed the experiments, A.R.L., L.T.W., and D.A. analyzed the data, and all authors contributed to the interpretation of results; M.H.S., B.H., T.V.L., T.U., I.M., O.A.A., and L.T.W. contributed materials, analysis tools or data; A.R.L., and D.A., wrote the paper and all authors contributed to critically revising the manuscript and gave their final approval of the submitted version.

**Conceived and designed the experiments:** A.R.L, K.K.K., L.T.W., M.H.S., and D.A.

**Performed the experiments:** A.R.L.

**Analyzed the data:** A.R.L., L.T.W., and D.A.

**Contributed to the interpretation of results:** All authors.

**Contributed materials, analysis tools or data:** B.H., T.V.L., T.U., I.M., O.A.A., and L.T.W.

**Wrote the manuscript:** A.R.L., and D.A.

**Contributed to critically revising the manuscript and gave their final approval of the submitted version:** All authors.

## Funding statement

This work was supported by Norsk forskningsråd [2018/14182], Helse sør-øst rhf [2019107, 2020086], Helse sør-øst rhf [2015044], Schweizerischer nationalfonds zur förderung der wissenschaftlichen forschung [PZ00P3_193658], Research Council of Norway [223273, 283799, 2837989], and European Research Council [ERC Starting Grant 802998].

## Data availability statement

Data associated with this study has been deposited https://osf.io/r64td/?view_only=8bee034ade5a46dc91b0e95654c2e780 and medrxiv: https://www.medrxiv.org/content/10.1101/2021.06.28.21259309v1, doi: https://doi.org/10.1101/2021.06.28.21259309.

## Declaration of interest's statement

The authors declare that they have no known competing financial interests or personal relationships that could have appeared to influence the work reported in this paper.
